# Machine Learning-Based Radiological Features and Diagnostic Predictive Model of Xanthogranulomatous Cholecystitis

**DOI:** 10.3389/fonc.2022.792077

**Published:** 2022-02-24

**Authors:** Qiao-Mei Zhou, Chuan-Xian Liu, Jia-Ping Zhou, Jie-Ni Yu, You Wang, Xiao-Jie Wang, Jian-Xia Xu, Ri-Sheng Yu

**Affiliations:** ^1^ Department of Radiology, The Second Affiliated Hospital, Zhejiang University School of Medicine, Hangzhou, China; ^2^ Department of Radiology, Jiaxing Hospital of Traditional Chinese Medicine, Jiaxing, China; ^3^ Department of Radiology, Sir Run Run Shaw Hospital, Zhejiang University School of Medicine, Hangzhou, China; ^4^ Department of Radiology, The Second Affiliated Hospital, Zhejiang Chinese Medical University, Hangzhou, China

**Keywords:** gallbladder cancer, radiological features, diagnostic prediction model, RandomForest, Xanthogranulomatous cholecystitis (XGC)

## Abstract

**Background:**

Xanthogranulomatous cholecystitis (XGC) is a rare benign chronic inflammatory disease of the gallbladder that is sometimes indistinguishable from gallbladder cancer (GBC), thereby affecting the decision of the choice of treatment. Thus, this study aimed to analyse the radiological characteristics of XGC and GBC to establish a diagnostic prediction model for differential diagnosis and clinical decision-making.

**Methods:**

We investigated radiological characteristics confirmed by the RandomForest and Logistic regression to establish computed tomography (CT), magnetic resonance imaging (MRI), CT/MRI models and diagnostic prediction model, and performed receiver operating characteristic curve (ROC) analysis to prove the effectiveness of the diagnostic prediction model.

**Results:**

Based on the optimal features confirmed by the RandomForest method, the mean area under the curve (AUC) of the ROC of the CT and MRI models was 0.817 (mean accuracy = 0.837) and 0.839 (mean accuracy = 0.842), respectively, whereas the CT/MRI model had a considerable predictive performance with the mean AUC of 0.897 (mean accuracy = 0.906). The diagnostic prediction model established for the convenience of clinical application was similar to the CT/MRI model with the mean AUC and accuracy of 0.888 and 0.898, respectively, indicating a preferable diagnostic efficiency in distinguishing XGC from GBC.

**Conclusions:**

The diagnostic prediction model showed good diagnostic accuracy for the preoperative discrimination of XGC and GBC, which might aid in clinical decision-making.

## Introduction

Xanthogranulomatous cholecystitis (XGC) is a rare and chronic inflammatory lesion of the gallbladder characterised by special types of granulomas and foamy cells (1). Epidemiological data from previous studies showed that the incidence of XGC was 1.3%–1.9% and that in India was 8.8%, with little difference in sex among regions (2). Goodman and Ishak described XGC as a special pathological entity in 1981 (3). Although XGC is benign, it is locally aggressive and may affect nearby organs such as the liver, duodenum, colon, and common bile duct (4). It may be misdiagnosed as gallbladder cancer (GBC) or may coexist with GBC because the inflammatory process involved resembles that of GBC in a macroscopic view.

The clinical manifestations and biological examination of XGC are nonspecific and similar to acute or chronic cholecystitis (5). Several studies have reported some imaging features that highly suggest XGC, including diffuse thickening of the gallbladder wall, continuous mucosal lines, intramural hypoattenuating nodules, and the presence of gallstones in the background of chronic gallbladder disease (6, 7). Approaches based on image analysis to guide the most appropriate treatment decisions have been intensively studied (8), and the applications of artificial intelligence (9), machine learning (10), and deep learning (11) in radiology images have been increasingly explored. Imaging tools such as computed tomography (CT) and magnetic resonance imaging (MRI) are generally utilised for clinical evaluation of gallbladder diseases, so analysis of their unique radiological features through machine learning may be a better method to distinguish XGC from GBC (12).

Laparoscopic cholecystectomy is the current gold standard for the treatment of benign gallbladder diseases. However, the high conversion rate of laparoscopic cholecystectomy to open cholecystectomy in XGC is related to the difficulty in distinguishing GBC (13). Moreover, surgeons may perform improper surgery owing to the misdiagnosis of GBC (14). In this study, we retrospectively analysed 60 cases of XGC and 93 cases of GBC confirmed by histopathology to establish a diagnostic prediction model for XGC and GBC based on CT and MRI features to help clinical decision-making on the appropriate treatment method for patients with XGC.

## Methods

### Patients

**Figure 1:**
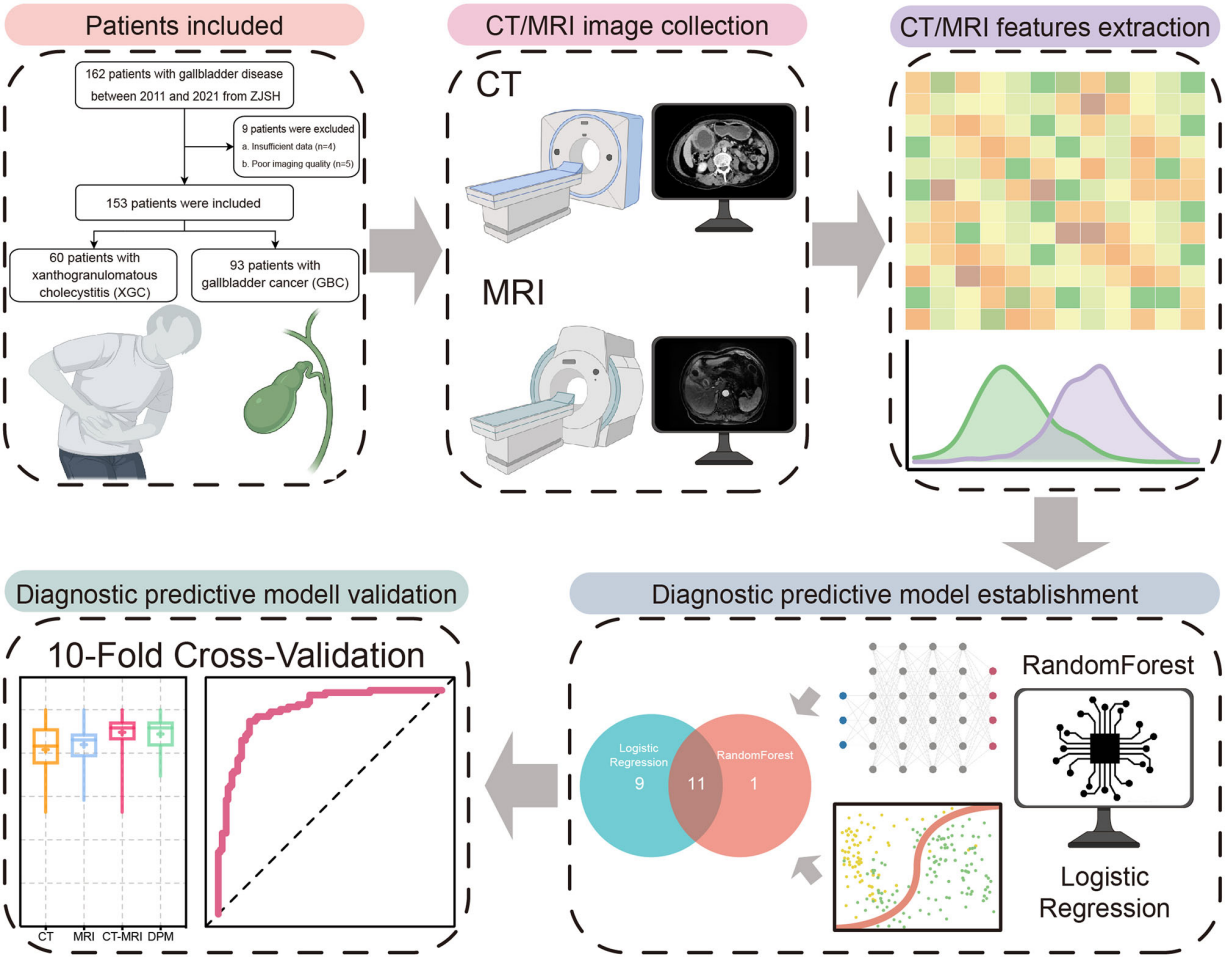
Schematic of research workflow and patient selection flowchart.

The flow chart of patient screening is presented in **Figure 1**. Patients with an inflammatory lesion originating in the gallbladder from the Second Affiliated Hospital of Zhejiang University School of Medicine from 2011 to 2021 and pathologically confirmed as XGC or GBC were included in the study population. These patients had satisfactory CT and MRI data and did not receive chemotherapy or radiotherapy. However, owing to limited data and poor imaging quality, some patients were excluded. The final study cohort comprised 60 patients with XGC and 93 patients with GBC. Our institutional review board approved this retrospective study and waived the requirement for patient consent.

### Image Acquisition

Multidetector-row CT (SOMATOM Definition Flash; Siemens Healthcare) was used to perform abdominal CT examinations. All patients underwent a plain scan under the fasting state initially and then received an injection of 100-mL contrast agent at 3-4 mL/s into the antecubital vein for enhanced scan. The arterial, portal vein, and equilibrium phases were obtained at 25, 60, and 100 s after the injection of the contrast agent.

A 3.0-T magnet (Signa or Discovery 750; GE Healthcare) with an eight-channel torso-phased array coil was used to obtain axial T1-weighted images (T1WI), T2-weighted images (T2WI), contrast-enhanced images and diffusion-weighted imaging (DWI) of all patients. Dynamic breath-hold imaging acquisitions were performed at 25–32, 45–52, 75–82, and 135–142 s after contrast enhancement to obtain the arterial, portal venous, equilibrium, and delayed phases. The imaging parameters of MRI were as follows: repetition time/echo time (TR/TE) of 205/3.2msec (or 3.9/1.8msec) and 6000/102.5msec (or 12000/86.3msec); the matrix of 256x256, the standard field of view of 4mm; the slice thickness of 4mm with no interslice gap. The parameters of DWI were as follows: TR/TE=1300/60.6msec or 6000/52.5 mec; water selective excitation for fat suppression; 5 mm thickness; matrix size of 128x128; field of view of 36x36cm; the number of shots of 6; slice thickness/gap of 5 mm/1.0 mm; 20 axial slices; scan time of 144s; b value of 0 and 600 s/mm2.

### Image Analysis

The original images were evaluated separately by two experienced abdominal radiologists who were blinded to patients’ pathological results. The variables of CT imaging were as follows: gallstones (no, gallbladder stones, intrahepatic bile duct stones, and extrahepatic bile duct stones), bile duct dilation (no, intrahepatic bile duct, extrahepatic bile duct, and intra and extrahepatic bile ducts), gallbladder cavity (normal, atrophy, and dilation), gallbladder morphology (regular and irregular), gallbladder wall thickness (≤10 and ≥10 mm), gallbladder wall thickening pattern (focal and diffuse), CT strengthening method (persistent, delayed, and other), intramural nodules (no, focal ≤20%, and diffuse >20%), intramural fat, mucosal lines (continuous, partly continuous, and disrupted), abnormal enhancement of adjacent liver parenchyma, liver/gallbladder interface (clear and blurred), fat around the gallbladder (clear and blurred), liver involvement, involvement of adjacent tissues outside the liver, peripheral lymph nodes (no, ≤10 mm, and >10 mm).

The variables of MRI were as follows: T1WI signal of the thickened cyst wall (high, equal, low, and mix), T2WI signal of the thickened cyst wall (high, equal, low, and mixed), MRI strengthening method (persistent, delayed, and other), T1WI signal of intramural nodules (no, high, equal, and low), T1WI signal of intramural nodules (no, high, equal, and low), lipid signal, bile signal, DWI (high, slightly high, and equal), apparent diffusion coefficient (ADC) (equal and low), and mucosal enhancement (no, obvious, and mild).

### Model Construction and Validation

The general technical workflow is presented in **Figure 1**. CT, MRI, and CT/MRI RandomForest (RF) classifiers were built based on the extracted CT and MRI image features. The “randomForest” package of R software (ver. 3.6.3; R Foundation for Statistical Computing, Vienna, Austria) was used to run the RF classifiers. Ten-fold cross-validation was performed to adjust the parameters of the classifiers and to determine the best diagnostic model during the training process. Univariate analysis was performed to evaluate the selected radiological features and their association with the above model. Finally, the most predictive parameters were used to construct the optimal diagnosis model using the RF method for distinguishing XGC from GBC.

### Statistical Analysis

SPSS ver. 26.0 (IBM Corp., Armonk, NY, USA), and R software (ver. 3.6.3; R Foundation for Statistical Computing, Vienna, Austria) were used to process all data. Continuous variables are presented as median with standard deviation and categorical variables as the number with a percentage. The same variables between the two groups were compared using the Mann–Whitney U-test for continuous non-normally distributed variables and chi-square or Fisher’s exact test for categorical variables. Variables that were statistically significant in the univariate analysis were obtained using ridge regression analysis to minimise multicollinearity, followed by the Logistic regression model. The models constructed using RF were validated using 10-fold cross-validation. Statistical significance was defined with a two-sided p-value of <0.05.

## Results

### Imaging Features

A comparison of CT and MRI features is summarised in **Table 1**. There were significant differences in the features of gallstones, bile duct dilation, gallbladder cavity, gallbladder morphology, gallbladder wall thickening pattern, strengthening method, intramural nodules, mucosal lines, abnormal enhancement of adjacent liver parenchyma, liver/gallbladder interface, fat around the gallbladder, liver involvement, and peripheral lymph nodes and T2WI signal of the thickened cyst wall, T1WI signal of intramural nodules, T2WI signal of intramural nodules, lipid signal, DWI, ADC, and mucosal enhancement.

**Table 1:** Imaging features in XGC and GBC patients.

Features	Xanthogranulomatous Cholecystitis (n = 60)		Gallbladder Cancer (n = 93)		P*
	n	%	n	%	
** CT Features**					
** Gallstones**					<0.001
No	4	6.7%	46	49.4%	
Gallbladder stones	54	90.0%	46	49.4%	
Intra-hepatic bile duct stones	1	1.7%	0	0.0%	
Extra-hepatic bile duct stones	1	1.7%	1	1.2%	
** Bile duct dilation**					<0.001
No	33	55.0%	53	57.0%	
Intra-hepatic bile duct	1	1.7%	17	18.3%	
Extra-hepatic bile duct	22	36.7%	8	8.6%	
Intrahepatic and extrahepatic bile duct	4	6.7%	15	16.1%	
** Gallbladder cavity**					0.021
Normal	3	5.0%	13	14.0%	
Atrophy	48	80.0%	76	81.7%	
Dilation	9	15.0%	4	4.3%	
** Gallbladder morphology**					0.002^†^
Regular	15	25.0%	6	6.5%	
Irregular	45	75.0%	87	93.5%	
** Gallbladder wall thickness**					0.407
≤ 10mm	15	25.0%	18	18.4%	
> 10mm	45	75.0%	75	80.6%	
** Gallbladder wall thickening pattern**					<0.001
Focal	14	23.3%	49	52.7%	
Diffuse	46	76.7%	44	47.3%	
** CT strengthening method**					0.012
Persistent	27	45.0%	61	65.6%	
Delay	31	51.7%	26	28.0%	
Other	2	3.3%	6	6.5%	
** Intramural nodules**					<0.001
No	21	35.0%	52	55.9%	
Focal (≤ 20%)	23	38.3%	36	38.7%	
Diffuse (> 20%)	16	26.7%	5	5.4%	
** Intramural fat**					0.177
No	42	70.0%	74	79.6%	
Yes	18	30.0%	19	20.4%	
** Mucosal lines**					<0.001
Continuous	20	33.3%	1	1.1%	
Partly continuous	28	46.7%	60	64.5%	
Disrupted	12	20.0%	32	34.4%	
** Abnormal enhancement of adjacent liver parenchyma**					0.001
No	11	18.3%	41	44.1%	
Yes	49	81.7%	52	55.9%	
** Liver/gallbladder interface**					0.008
Clear	15	25.0%	43	46.2%	
Blurred	45	75.0%	50	53.8%	
** Fat around the gallbladder**					0.001
Clear	13	21.7%	46	49.5%	
Blurred	47	78.3%	47	50.5%	
** Liver involvement**					0.004
No	46	76.7%	50	53.8%	
Yes	14	23.3%	43	46.2%	
** Involve adjacent tissues outside the liver**					0.927
No	26	43.3%	41	44.1%	
Yes	34	56.7%	52	55.9%	
** Peripheral lymph nodes**					0.001
No	44	73.3.0%	42	45.2%	
≤ 10 mm	13	21.7%	27	29.0%	
> 10mm	3	5.0%	24	25.8%	
** MRI Features**					
** T1WI signal of thickened cyst wall**					0.085
High	3	5.0%	4	4.3%	
Equal	22	36.7%	41	44.1%	
Low	23	38.3%	42	45.2%	
Mix	12	20.0%	6	6.5%	
**T2WI signal of thickened cyst wall**					0.001
High	5	8.3%	14	15.1%	
Equal	37	61.7%	48	51.6%	
Low	7	11.7%	28	30.1%	
Mix	11	18.3%	3	3.2%	
** MRI strengthening method**					0.725
Persistent	17	28.3%	28	30.1%	
Delay	37	61.7%	59	63.4%	
Other	6	10.0%	6	6.5%	
**T1WI signal of intramural nodules**					<0.001
No	5	8.3%	68	73.1%	
High	10	16.7%	3	3.2%	
Equal	24	40.0%	14	15.1%	
Low	21	35.0%	8	8.6%	
**T2WI signal of intramural nodules**					<0.001
No	5	8.3%	68	73.1%	
High	38	63.3%	20	21.5%	
Equal	15	25.0%	5	5.4%	
Low	2	3.4%	0	0.0%	
**Lipid signal**					<0.001
No	22	36.7%	82	88.2%	
Yes	38	63.3%	11	11.8%	
** Bile signal**					0.218
No	42	70.0%	56	60.2%	
Yes	18	30.0%	37	39.8%	
** DWI**					0.004
High	21	35.0%	58	62.4%	
Slightly high	30	50.0%	27	29.0%	
Equal	9	15.0%	8	8.6%	
ADC					<0.001
Equal	44	73.3%	30	32.3%	
Low	16	26.7%	63	67.7%	
** Mucosal enhancement**					<0.001
No	6	10.0%	39	41.9%	
Obvious	39	65.0%	21	22.6%	
Mild	15	25.0%	33	35.5%	

*Calculated by Chi-Square test; ^†^calculated by Fisher exact test.

### RF Model

**Figure 2:**
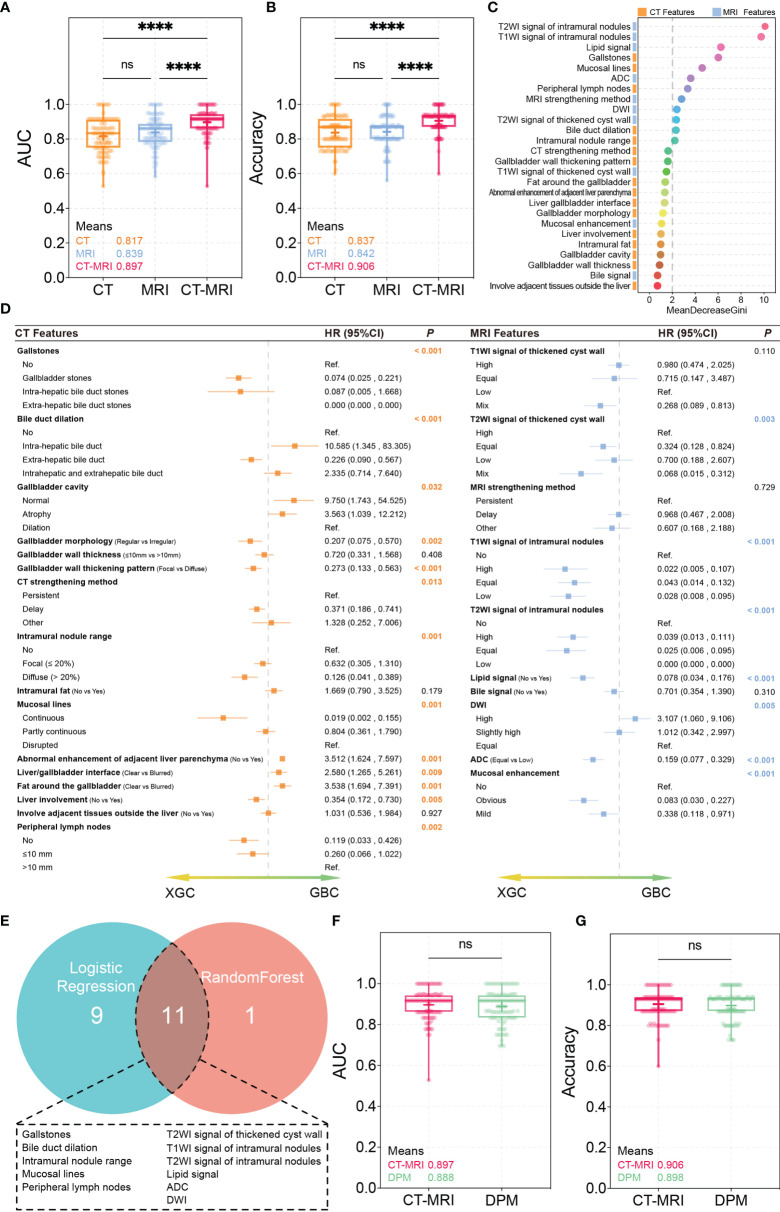
RandomForest and Logistic regression test results. **(A, B)** AUC and accuracy of the CT, MRI, CT/MRI models. ****P < 0.0001; ns, not significant, Mann-Whitney U Test. **(C)** The importance of variables measured using the CT/MRI model with the Random Forest Gini Index. **(D)** Logistic regression for univariate analysis. **(E)** Correlation of variables obtained using random forest and logistic regression. **(F, G)** AUC and accuracy of the CT/MRI model and diagnosis prediction model. ns, not significant, Mann-Whitney U Test.

Based on the characteristics of the collected CT and MRI scans, the RF method was performed to establish three differential diagnosis models of XGC and GBC. In the training process, 10-fold cross-validation was performed 10 times to adjust the parameters of the classifier. Performance evaluation was conducted using the mean AUC. The AUCs of the CT and MRI models were 0.817 and 0.839, respectively, with corresponding accuracies of 0.837 and 0.842. The CT/MRI model showed good discrimination between XGC and GBC with an AUC of 0.897. Additionally, the CT/MRI model showed a high diagnostic performance with an accuracy of 0.906 (**Figures 2A, B**), thereby confirming the diagnostic importance of radiological features for the recognition of XGC. Furthermore, the Gini index can be used to judge the importance of different variables in the RF model (**Figure 2C**).

### Diagnostic Predictive Model

A total of 20 imaging features showed statistical differences in the univariate analysis through Logistic regression (**Figure 2D**). To lapply the above diagnostic system conveniently in practice, important imaging features extracted by the two machine learning methods were integrated to further build a new differential diagnosis model. A total of 11 imaging features were retained, including those of gallstones, bile duct dilation, intramural nodules, mucosal lines, and peripheral lymph nodes and T2WI signal of the thickened cyst wall, T1WI signal of intramural nodules, T2WI signal of intramural nodules, lipid signal, ADC, and DWI (**Figure 2E**).

Subsequently, we constructed a diagnostic predictive model using these 11 parameters using the RF method. Ten times of 10-fold cross-validation was performed to evaluate the reliability and reproducibility of the diagnostic predictive model. Finally, the average AUC and accuracy of the diagnostic prediction model were 0.888 and 0.898, respectively, which are similar to those of the primary CT/MRI model (**Figures 2F, G**). Interestingly, the comparison of ROCs verified using the Mann–Whitney U-test showed no statistical difference between the two models, indicating that the diagnostic predictive model made full use of the value of the CT/MRI model. These features were ranked according to their relevance to the differential diagnosis as follows: T2WI signal of intramural nodules, T1WI signal of intramural nodules, lipid signal, gallstones, mucosal lines, ADC, peripheral lymph nodes, DWI, T2WI signal of the thickened cyst wall, bile duct dilation, and intramural nodules.

## Discussion

XGC was originally considered a malignant disease process; however, many studies have now confirmed its benign course (15), which is characterised by a focal or diffuse destructive inflammatory process, followed by significant macrophage and foam cell infiltrations and hyperplastic fibrosis. Concerning the pathogenesis of XGC, the most widely accepted theory is that the extravasation of bile through the ruptured Rokitansky-Aschoff sinus or increased internal pressure of the biliary tract causes small mucosal ulcers (16). Extensive inflammatory fibrosis results in the thickening of the gallbladder wall and the formation of multiple yellowish-brown nodules (17). The inflammatory process often spreads to adjacent organs, thereby forming a tumour-like mass around the gallbladder (18). Since XGC is often misdiagnosed as GBC, which leads to unnecessary radical surgical excision, it is important to distinguish between XGC and GBC before surgery.

Although ultrasound is a clinically preferred and ubiquitous imaging method, its specificity for XGC is low (12). Clinically, CT and MRI are widely used to distinguish between XGC and GBC because of their convenience and non-invasiveness (19). We reviewed a large amount of literature in the early stages and incorporated the reported potential radiological features that may distinguish XGC from GBC into the initial model. Using the RF algorithm and Logistic regression method, it was shown that the diagnostic prediction model had a stronger ability to distinguish between XGC and GBC with an AUC of 0.888 and an accuracy of 0.898. This model, constructed with outstanding clinical applicability, summarised 11 radiological features and had high diagnostic performance, thereby confirming the importance of imaging features in the differential diagnosis of XGC and GBC.

**Figure 3:**
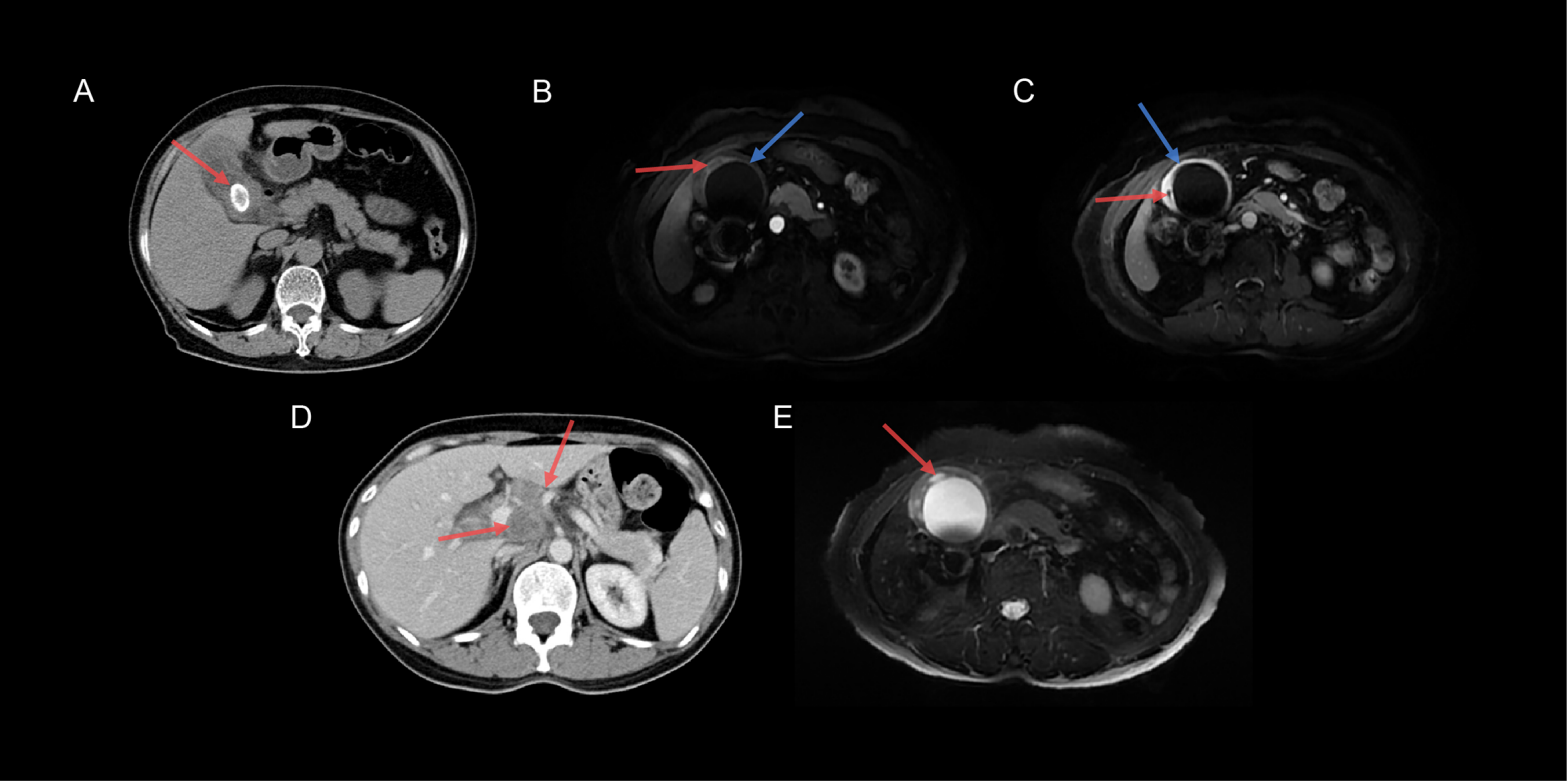
Typical images of XGC and GBC specimens. **(A)** The CT transverse section revealed cholecystolithiasis (red arrow) in a 46-year-old women with XGC. **(B)** Axial T1-weighted MRI-enhanced image showed low-intensity nodules (red arrow) in the gallbladder wall, and the continuous mucosal line was significantly enhanced (blue arrow) in the arterial phase. **(C)** Axial T1-weighted MRI-enhanced image showed that the gallbladder wall was obviously enhanced; however, the intramural nodules were unenhanced (red arrow), and the mucosal line was intact (blue arrow) in the delayed phase. **(D)** Axial CT image demonstrated two lymph nodes (>10 mm) in the hilar (red arrow) in a 46-year-old women with GBC. **(E)** Axial T2-weighted MRI-enhanced image demonstrated that the gallbladder wall was apparently thickened with multiple high-intensity nodules (red arrow).

CT findings included gallstones, mucosal lines, peripheral lymph nodes, bile duct dilation, and intramural nodules. Studies have shown that the development of XGC may be related to gallstones (17, 20), similar to our findings (**Figure 3A**). Additionally, intramural hypodense nodules, continuous mucosal lines, and absence of intrahepatic bile duct dilatation suggested XGC (**Figures 3B, C**). Goshima et al. reported that three of the five CT features that meet the XGC characteristics can provide high accuracy in distinguishing XGC from GBC (21), which is consistent with our results. Peripheral lymphadenopathy (>10 mm) was an independent factor in the diagnosis of GBC (**Figure 3D**), which may help in the correct preoperative diagnosis of XGC. As for the MRI features, the high-signal area of the T2WI corresponded to the area of significant xanthogranulomas (**Figure 3E**), which is consistent with a previous finding (22). The lipid signal of XGC nodules was owing to tissue cell phagocytosis and accumulation of extravasated bile and bile lipids. Ogawa et al. showed that the positive signal rate with DWI was significantly higher in GBC than in benign gallbladder diseases (23). DWI and ADC may play a significant role in distinguishing XGC from GBC because they are beneficial for distinguishing malignant and benign lesions.

XGC should be operated immediately once diagnosed to prevent complications owing to its invasiveness and destructiveness (24). Compared with open surgery, laparoscopic cholecystectomy has a shorter operating time and shorter postoperative hospital stay (25), which indicates that in some strictly selected cases, laparoscopic surgery is superior to open surgery for XGC treatment (4). Our predictive diagnosis model can distinguish XGC from GBC through adequate preoperative evaluation, helps clinical decision-making, and reduces the incidence of postoperative complications. Despite the high accuracy of the predictive diagnosis model, intraoperative diagnosis is still necessary because XGC may coexist with GBC. The frequency of coexistence of these two lesions was approximately 19.6% in India (26). However, there was no coexistence of XGC and GBC in our study, which is one of the limitations of this study. Owing to the retrospective study design, patients with only a single examination and obvious imaging findings were not included, so there may be an inherent selection bias. On the other hand, there were individual variables that have reported differences; however, they were not significant in our study cohort, indicating that more patients may need to be introduced in future studies.

## Conclusion

We established a diagnostic prediction model for XGC using the RF method and logistic regression. The model contains 11 important critical variables, including T2WI signal of intramural nodules, T1WI signal of intramural nodules, lipid signal, gallstones, mucosal lines, ADC, peripheral lymph nodes, DWI, T2WI signal of the thickened cyst wall, bile duct dilation, and intramural nodules. This diagnostic prediction model has exhibited good discrimination between XGC and GBC, thus providing significant assistance in clinical practice.

## Data Availability Statement

The raw data supporting the conclusions of this article will be made available by the authors, without undue reservation.

## Ethics Statement

The studies involving human participants were reviewed and approved by The Second Affiliated Hospital of Zhejiang University School of Medicine (Approval numbers 20210806). Written informed consent for participation was not required for this study in accordance with the national legislation and the institutional requirements.

## Author Contributions

QZ conceived the study and drafted the manuscript. Ri-Sheng Yu and JX helped critically revise the manuscript for important intellectual content. QZ, CL and JZ performed the research. JY, YW and XW helped analyse data. All authors contributed to the article and approved the submitted version.

## Funding

This study was supported by grants from the Zhejiang Medical and Health Science and Technology Plan Project (Grant Numbers 2019KY476).

## Conflict of Interest

The authors declare that the research was conducted in the absence of any commercial or financial relationships that could be construed as a potential conflict of interest.

## Publisher’s Note

All claims expressed in this article are solely those of the authors and do not necessarily represent those of their affiliated organizations, or those of the publisher, the editors and the reviewers. Any product that may be evaluated in this article, or claim that may be made by its manufacturer, is not guaranteed or endorsed by the publisher.

## Abbreviations

XGC, xanthogranulomatous cholecystitis; GBC, gallbladder cancer; CT, computed tomography; MRI, magnetic resonance imaging; RF, Random Forest; AUC, the area under the curve; ROC, the receiver operating characteristic curve; T1WI, T1 weighted imaging; T2WI, T1 weighted imaging; DWI, diffusion-weighted magnetic resonance imaging; ADC, apparent diffusion coefficient.
